# An analysis of paediatric clinical presentations in Northwest Syria and the effect of forced displacement, 2018–2022

**DOI:** 10.1016/j.gloepi.2024.100146

**Published:** 2024-06-04

**Authors:** Vinay Kampalath, Ms Maia C. Tarnas, Ms Vaibhavee Patel, Mohamed Hamze, Randa Loutfi, Bachir Tajaldin, Ahmad Albik, Ayman Kassas, Anas Khashata, Aula Abbara

**Affiliations:** a Perelman School of Medicine, University of Pennsylvania, USA; b Department of Population Health and Disease Prevention, University of California Irvine, USA; c Imperial College, London, UK; d Syrian American Medical Society, Washington, DC, USA

## Abstract

**Background:**

One in six children worldwide lives in a region exposed to armed conflict. In conflicts, children are among the most vulnerable, and at risk of adverse health outcomes. We sought to describe trends in child and adolescent morbidity in northwest Syria (NWS) and understand how forced displacement affects clinical utilisation during the Syrian conflict.

**Methods:**

Retrospective data between January 2018 and December 2022 were obtained from the Syrian American Medical Society (SAMS), a non-governmental organisation that operates health facilities in NWS. After initial descriptive analyses were completed, we performed a seasonal-trend decomposition to estimate the seasonality of clinical presentations. We subsequently employed a multivariate regression model incorporating age, gender, residency status, season, and a random district-level intercept to measure the association between the odds of clinical consultation and forced displacement.

**Findings:**

Across 51 reporting SAMS facilities, 2,687,807 clinical consultations were studied over a five-year period. Seasonality was demonstrated for every clinical consultation category. Higher levels of forced displacement were associated with increased odds of consultations for nutrition, trauma, NCDs and mental health and decreased odds of consultation for communicable diseases. Aside from traumatic injury, internally displaced persons (IDPs) had higher AORs of clinical consultations compared to host populations.

**Interpretation:**

Forced displacement differentially impacts clinical utilisation among children in northwest Syria, and the effects of displacement persist for at least six months. Clinical needs vary by host/IDP status, sex, age, and season. This study can assist policymakers in forecasting the health needs of children in northwest Syria.

## Introduction

Conflict often disproportionately affects the health of children and adolescents [1]. As of 2022, around 449 million children, or one in six globally, live in areas of armed conflict [2]. Exposure to conflict increases morbidity and mortality in children, with neonates (children ≤28 days) facing the largest impacts [3]. These effects can occur directly, such as through conflict-related injuries, and indirectly through increases in communicable diseases and malnutrition due to conflict-related changes in the natural and built environments [4,5]. Such changes include the proliferation of disease vectors, degradation of critical infrastructure, attacks on healthcare, and fragmentation of regional health systems [6,7]. Exposure to conflict in childhood produces long-term health complications, including physical disability, impaired development from toxic stress, and mental health disorders [7]. Conflict and forced displacement fundamentally change children's social determinants of health, leading to financial insecurity, disruption in social networks, and interruptions to education [1,5].

These consequences have been profound in Syria, where protracted conflict is now in its twelfth year. Over 14 million Syrians have been forcibly displaced, including 6.8 million internally displaced people (IDPs) [8]. Around half of these IDPs are children. Over 30,000 children have died directly from conflict, and thousands more face the impacts of conflict on the health system [9]. Ninety per cent of the population live below the poverty line, and 6.5 million children require humanitarian assistance, with 4.6 million facing food insecurity [10]. Chronic underfunding has allowed this situation to deteriorate. Closures of border crossings, attacks on healthcare facilities, and the February 2023 earthquakes on the Türkiye/Syria border have further complicated humanitarian aid efforts. Cycles of acute emergencies, longstanding underfunding in aid, and unequal aid distribution have led to the continued prioritisation of emergency services rather than comprehensive and preventive care [11].

Though all of Syria has been affected by conflict, northwest Syria (NWS) has experienced staggering devastation. Over 65% of the 4.5 million people in NWS are IDPs, of which 1.5 million are children [12]. Children in NWS face particularly dire overcrowding, unsanitary living conditions, limited access to clean water and basic necessities, and barriers to accessing healthcare [13]. To date, there is little data on paediatric healthcare utilisation in conflict settings, particularly in NWS. A 2022 scoping review noted the high prevalence of traumatic injuries, communicable diseases, chronic malnutrition, and mental illness among Syrian children [4]. That review also noted the dearth of academic literature, particularly from recent years of the conflict. In this paper, we aim to address this gap by analysing paediatric clinical consultation data from NWS between January 2018 and December 2022, including potential associations with forced displacement.

## Methods

### Design and setting

This retrospective quantitative analysis uses clinical consultation data from Syrian American Medical Society (SAMS) facilities in NWS between January 2018 and December 2022. SAMS provides primary-, secondary-, and tertiary-level care in NWS, along with mobile clinics and ambulance services which can be reached by around 70% of the population in this area. NWS currently comprises most of Idlib governorate and parts of Aleppo governorate, which are outside government control. During the beginning of the study period, Idlib and Aleppo witnessed significant population movement. Forced displacement peaked between December 2019 and March 2020, with nearly one million people displaced due to violence, 60% of whom were children [14]. During this time, repeated targeted attacks on healthcare workers and facilities led to facility closures in southern Idlib governorate.^24^ Because of the fragmented health system in the region, local and international humanitarian organisations are the predominant providers of healthcare services.

**Fig. 1:**
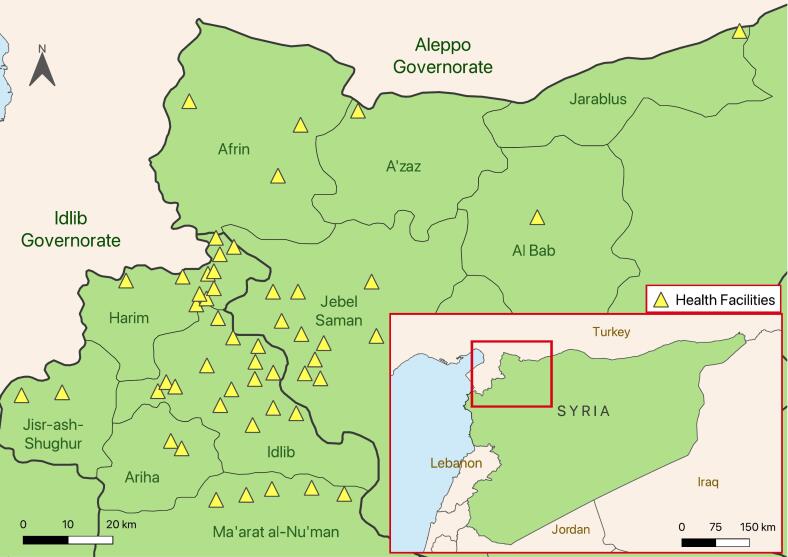
Map of study area. Health facility locations are approximate.

We included data from 51 facilities in 10 districts, though most were not operational during the entirety of the study period (Fig. 1). The majority (69%, 35/51) of facilities are located in Idlib governorate, with most in Idlib (*n* = 15) and Harim (*n* = 11) districts. Of the 16 facilities in Aleppo governorate, ten were located in Jebel Shaman district.

### Data sources

SAMS provided de-identified data on all paediatric (under 18) consultations at SAMS facilities in the study area between January 2018 and December 2022 in Arabic. These account for around 15% of all clinical consultations in NWS during the study period [15]. Data on facility name and identification number, district, date of visit, age at visit, gender, residency status (IDP or host), ICD-10 diagnosis, procedures completed (if any), and visit outcome were included.

We translated the data into English and extracted consultations related to vaccine-preventable diseases (VPD), other infectious diseases (INF), noncommunicable diseases (NCD), trauma, injury, and poisoning (TRA), mental and psychosocial health (MBD), and nutrition (NTR) based on ICD-10 diagnosis codes. We focused on these consultation types as they captured the majority of consultations and were not sex- or age-specific. These categories were reduced to 1) VPD & INF, 2) NTR, 3) TRA, and 4) NCD & MBD to account for similarities in pathology and approaches to treatment. This categorisation process was informed by two consultant-level physicians on the research team. Ages were aggregated into categories of <1 year, 2–4 years, 5–11 years, and 12–17 years. We also assigned seasonal categories to each consultation because of expected seasonal variation. These were defined using the World Bank's Climate Change Knowledge Portal and were Winter (December–February), Spring (March–May), Summer (June–August), and Autumn (September–November).

We also extracted governorate-level data on IDP movements in Idlib and Aleppo from the Office for the Coordination of Humanitarian Affairs (OCHA) Türkiye [16]. We aggregated data from both governorates and created monthly severity categories based on quartiles. For the unlagged data, these categories were: Low (≤26,330 monthly IDP movements), Medium (26,331–41,936), High (41,937–82,051), and Severe (≥82,052) displacement. To assess the effects of displacement on clinical consultations over time, we experimented with several different lags for displacement and readjusted the category bounds appropriately. Briefly, this means that IDP movements in a given month were used to predict consultations in subsequent months (varying based on the length of the lag).

### Statistical analysis

To assess trends in consultations amid seasonal fluctuations, we utilised seasonal-trend decomposition using locally estimated scatterplot smoothing. This decomposes a time series into three components: 1) seasonal, 2) trend, or long-term change over the time series, and 3) remaining variation [17]. We also used multivariable logistic regression with a random district-level intercept to measure associations between each consultation category and forced displacement while controlling for patient age, gender, residency status, and season. The random intercept accounts for variation in baseline risk across districts. We compared these models to ones without the intercept using various model fit statistics and found that the mixed model was a better fit (appendix p.1–2). For each of the four consultation categories, we ran a model with no displacement lag, 1-month, 2-month, and 6-month lags to assess any potential changes in the adjusted odds ratios (AOR). Unadjusted odds ratios can be found in the appendix (p.3). All analyses were conducted in R v4.0.4.

### Ethical considerations

SAMS anonymised all data before review by the study team. Ethics approval was granted by Imperial College London's research governance and integrity team (reference number: 6533963). The SAMS research committee also approved the study.

### Role of funding source

There was no funding source for this study.

## Results

### General trends

There were 3,185,640 total paediatric consultations conducted at 51 SAMS facilities in NWS between January 2018 and December 2022. We included 2,687,807 in our sample based on consultation category (Table 1); the remaining consultations comprise other clinical categories not included in this study. 2020 had the lowest patient volume (*n* = 448,870), which coincides with a peak in forced displacement in early 2020 and regional restrictions on healthcare access and physical movement during the first wave of the COVID-19 pandemic.^56^ SAMS facilities averaged 559,734 VPD-, INF-, NTR-, TRA- NCD-, and MBD-related consultations annually for the other four study years. VPD & INF consultations accounted for 56.6% of all clinical visits over the study period.

**Table 1:** Summary of consultations per category. Cumulative percentages may not total 100% due to rounding.

	**VPD & INF**		**NTR**		**TRA**		**NCD & MBD**		**Total**	
	No.	%	No.	%	No.	%	No.	%	No.	%
**Total Consultations**	**1,521,978**	**56.6%**	**20,982**	**0.78%**	**359,284**	**13.4%**	**785,563**	**29.2%**	**2,687,807**	**100%**
**Year**										
2018	318,939	(21%)	3577	(17%)	66,797	(19%)	151,689	(19%)	541,002	(20%)
2019	317,119	(21%)	4898	(23%)	84,681	(24%)	175,268	(22%)	581,966	(22%)
2020	260,864	(17%)	4479	(21%)	56,375	(16%)	127,152	(16%)	448,870	(17%)
2021	312,041	(21%)	4284	(20%)	78,641	(22%)	174,018	(22%)	568,984	(21%)
2022	313,015	(21%)	3744	(18%)	72,790	(20%)	157,436	(20%)	546,985	(20%)

**Residency**										
Host	627,044	(41%)	8236	(39%)	192,846	(54%)	331,802	(42%)	1,159,928	(43%)
Displaced	894,934	(59%)	12,746	(61%)	166,438	(46%)	453,761	(58%)	1,527,879	(57%)

**Age (yr.)**										
0–1	310,766	(20%)	6368	(30%)	11,484	(3%)	109,852	(14%)	438,470	(16%)
2–4	647,563	(43%)	9677	(46%)	104,158	(29%)	261,973	(33%)	1,023,371	(38%)
5–11	420,510	(28%)	3436	(16%)	128,904	(36%)	238,652	(30%)	791,502	(29%)
12–17	143,139	(9%)	1501	(7%)	114,738	(32%)	175,086	(22%)	434,464	(16%)

**Sex**										
Male	833,118	(55%)	9406	(45%)	243,580	(68%)	406,785	(52%)	1,492,889	(56%)
Female	688,860	(45%)	11,576	(55%)	115,704	(32%)	378,778	(48%)	1,194,918	(44%)

Children under five years comprised most consultations (54%), as did males (56%). In 2018 and 2019, most consultations were for host children (56%), though this inverted following the peak displacement period between December 2019 and February 2020. Between 2020 and 2022, IDP children constituted 67% of consultations. This difference was most pronounced in 2022 when the ratio for IDP to host children consultations was over 2:1.

Health facilities in Jarablus (*n* = 1,005,947), Harim (*n* = 597,509), and Jebel Saman (*n* = 386,885) reported the highest number of consultations over the study period, but Ariha (*n* = 70,299), Jarablus (*n* = 67,063), and Afrin (*n* = 65,220) reported the highest number of consultations per health facility. Within each district, there was notable fluctuation in the number of reported consultations by year, though there was likewise fluctuation in the number of reporting facilities each year.

### Seasonality

**Fig. 2:**
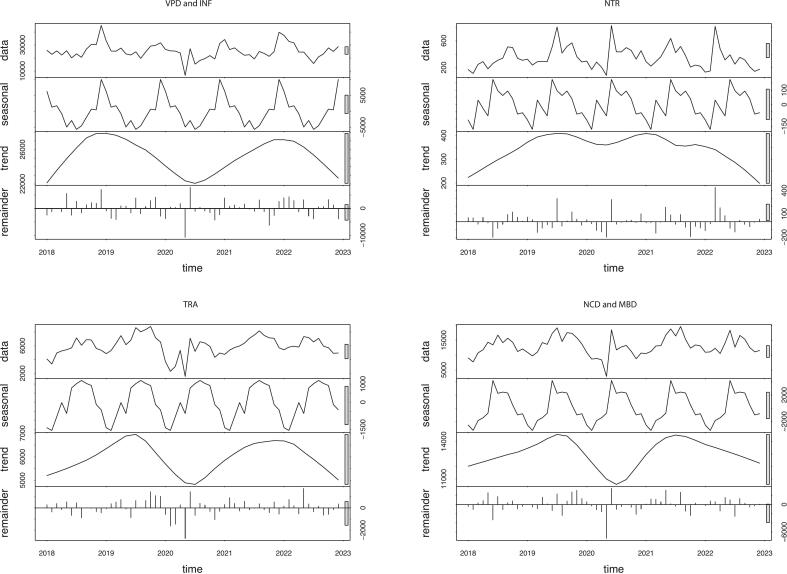
Seasonal trend decomposition with LOESS (Locally Estimated Scatterplot Smoothing) for each consultation category. The rows in each graph are: 1) data, the complete time series, 2) seasonal, the seasonal pattern; 3) trend, the long-term change over the period; and 4) remainder. VPD: vaccine-preventable diseases; INF: other infectious diseases; NTR: nutrition; TRA: trauma, injury, and poisoning; NCD: non-communicable diseases; MBD: mental and psychosocial health.

Seasonal variation was noted in all consultation categories. VPD & INF consultations peaked in the winter, whereas all others peaked in the summer. Following seasonal trend decomposition, VPD & INF, TRA, and NCD & MBD consultations had bimodal trends throughout the study period, with consultations reaching a nadir in mid-2020 (Fig. 2). NTR consultations had minor fluctuations around this time but remained high.

### Multivariable regression

Each consultation category was differentially associated with the covariates of interest (Table 2). Displacement increased consultation odds across each severity level for NTR, TRA, and NCD & MBD, and decreased the odds for VPD & INF consultations. Compared to medium and high displacement levels, severe displacement generally had stronger associations with each outcome. IDPs had higher odds of a VPD & INF, NTR, and NCD & MBD consultation when compared to individuals from the host community. This was particularly pronounced in VPD & INF and NTR consultations, where being an IDP increased the odds of consultation by 23% (AOR: 1.23, 95% CI: 1.22–1.24) and 27% (AOR: 1.27, 95% CI: 1.23–1.31), respectively. IDP status reduced the odds of a TRA consultation by 35% (AOR: 0.65, 95% CI: 0.64–0.65).

**Table 2:** AORs (Adjusted Odds Ratios) of consultation by category. Displacement is not lagged. VPD: vaccine-preventable diseases; INF: other infectious diseases; NTR: nutrition; TRA: trauma, injury, and poisoning; NCD: noncommunicable diseases; MBD: mental and psychosocial health.

	**VPD & INF**	**NTR**	**TRA**	**NCD & MBD**
	*OR (95% CI)*	*OR (95% CI)*	*OR (95% CI)*	*OR (95% CI)*
**Displacement**				
Low	Reference			
Medium	0.83 (0.82–0.84)	1.31 (1.24–1.37)	1.20 (1.18–1.21)	1.10 (1.09–1.11)
High	0.85 (0.84–0.86)	1.70 (1.61–1.80)	1.15 (1.13–1.17)	1.11 (1.10–1.13)
Severe	0.81 (0.80–0.82)	1.55 (1.46–1.66)	1.21 (1.19–1.23)	1.19 (1.18–1.20)

**Residency**				
Host	Reference			
Displaced	1.23 (1.22–1.24)	1.27 (1.23–1.31)	0.65 (0.64–0.65)	1.05 (1.04–1.06)

**Age (yr.)**				
0–1	Reference			
2–4	1.26 (1.26–1.27)	0.84 (0.82–0.87)	5.21 (5.11–5.32)	1.37 (1.35–1.38)
5–11	0.83 (0.83–0.84)	0.39 (0.37–0.40)	8.47 (8.31–8.64)	1.65 (1.64–1.67)
12–17	0.31 (0.30–0.31)	0.26 (0.25–0.28)	12.33 (12.09–12.57)	1.94 (1.93–1.96)

**Sex**				
Male	Reference			
Female	1.00 (1.00–1.01)	1.52 (1.48–1.56)	0.50 (0.49–0.50)	1.14 (1.14–1.15)

**Season**				
Winter	Reference			
Spring	0.73 (0.72–0.73)	1.51 (1.45–1.58)	1.26 (1.24–1.27)	1.25 (1.24–1.26)
Summer	0.48 (0.48–0.48)	1.93 (1.85–2.01)	1.47 (1.45–1.48)	1.70 (1.69–1.72)
Autumn	0.62 (0.62–0.63)	1.56 (1.50–1.63)	1.41 (1.40–1.43)	1.37 (1.36–1.38)
**Temporality**	Reference			
Year	0.92 (0.91–0.92)	1.05 (1.03–1.07)	1.14 (1.14–1.15)	1.03 (1.03–1.03)

Adolescents (ages 12–17 years) had the highest odds of a TRA (AOR: 12.33, 95% CI: 12.09–12.57) and NCD & MBD (AOR: 1.94, 95% CI: 1.93–1.96) consultation, and the odds of consultations for these categories decreased with age. Children between 2 and 4 years had the highest odds of a VPD & INF consultation (AOR 1.26, 95% CI: 1.26–1.27). NTR-related consultations were most likely to occur in children under one year, and the odds reduced with increasing age. Girls had 52% higher odds (AOR: 1.52, 95% CI: 1.48–1.56) of having an NTR consultation and 14% higher odds (AOR: 1.14, 95% CI: 1.14–1.15) of an NCD & MBD consultation than boys. Conversely, boys had higher odds of a TRA consultation. There were no differences by sex for VPD & INF consultations.

### Displacement effects over time

Displacement was associated with consultation odds in each category, and these associations persisted six months beyond the displacement event (Table 3). The mixed models in Table 3 were adjusted for residency status, age, sex, season, and year, though we only show the displacement outputs for ease of interpretability (full outputs in appendix p.4–5). AORs generally moved towards one with increasing time lags (except for NTR). NTR consultations were most strongly associated with displacement, with periods of high displacement having 70% higher odds (AOR: 1.70; 95% CI: 1.61–1.80) of a consultation compared with periods of low displacement. Six months following the displacement event, odds were still increased by 60% (AOR: 1.60; 95% CI: 1.50–1.70).

**Table 3:** AORs (Adjusted Odds Ratios) of multivariable logistic regression for each consultation category at varying displacement lags. Each model is fully adjusted, but only the displacement results are shown. VPD: vaccine-preventable diseases; INF: other infectious diseases; NTR: nutrition; TRA: trauma, injury, and poisoning; NCD: noncommunicable diseases; MBD: mental and psychosocial health.

	**VPD & INF**	**NTR**	**TRA**	**NCD & MBD**
	*OR (95% CI)*	*OR (95% CI)*	*OR (95% CI)*	*OR (95% CI)*
**Displacement**	**No Lag**			
Low	Reference			
Medium	0.83 (0.82–0.84)	1.31 (1.24–1.37)	1.20 (1.18–1.21)	1.10 (1.09–1.11)
High	0.85 (0.84–0.86)	1.70 (1.61–1.80)	1.15 (1.13–1.17)	1.11 (1.10–1.13)
Severe	0.81 (0.80–0.82)	1.55 (1.46–1.66)	1.21 (1.19–1.23)	1.19 (1.18–1.20)
**Displacement**	**1 Month Lag**			
Low	Reference			
Medium	0.81 (0.80–0.81)	1.43 (1.37–1.50)	1.18 (1.17–1.20)	1.14 (1.13–1.15)
High	0.85 (0.84–0.86)	1.56 (1.47–1.65)	1.23 (1.21–1.25)	1.08 (1.06–1.09)
Severe	0.85 (0.84–0.85)	1.51 (1.42–1.61)	1.19 (1.17–1.21)	1.15 (1.14–1.16)
**Displacement**	**6 Month Lag**			
Low	Reference			
Medium	0.87 (0.87–0.88)	1.36 (1.30–1.43)	1.11 (1.09–1.12)	1.08 (1.07–1.09)
High	0.88 (0.87–0.89)	1.60 (1.50–1.70)	1.23 (1.21–1.25)	1.04 (1.03–1.05)
Severe	0.86 (0.86–0.87)	1.57 (1.48–1.66)	1.15 (1.13–1.17)	1.08 (1.07–1.10)

Increased odds also persisted for TRA and NCD & MBD consultations. Immediately following displacement, the odds of a TRA consultation were 15–21% higher when compared to periods with low displacement. Odds continued to be 11–23% higher six months following the displacement event. Similarly, odds for an NCD & MBD consultation continued to be elevated six months following a displacement event (4–8%), though these effects were reduced compared to more immediate associations. Conversely, displacement was associated with reduced odds of VPD & INF consultations. Severe displacement reduced the odds of a VPD & INF consultation by 19% (AOR: 0.81; 95% CI: 0.80–0.82) in the same month. Six months following the event, the odds were still reduced by 14% (AOR: 0.86; 95% CI: 0.86–0.87).

For each consultation category other than TRA, displaced children had increased odds of consultation when compared to host children (appendix p.4–5). This was most pronounced for VPD & INF and NTR consultations and persisted across each lag. Host children, however, had higher odds of a trauma-related consultation across each lag.

## Discussion

Representing nearly 2.7 million clinical consultations over five years, this study is one of the largest analyses of clinical utilisation in a humanitarian setting; to our knowledge, this is also the most extensive analysis of paediatric health utilisation in a conflict setting [5]. Importantly, it includes an analysis of paediatric NCDs and mental health, which are underappreciated in humanitarian settings. This study reveals that several factors, including age, sex, season, residency status, and forced displacement impact paediatric clinical utilisation in NWS. The effects of displacement are nuanced insofar as they vary by the magnitude of displacement and the type of consultation.

This study demonstrated seasonal trends in clinical utilisation that persisted even through the COVID-19 pandemic. Seasonal variation in the epidemiology of communicable diseases, malnutrition, injury, NCDs, and mental illness among children has been well documented in several countries [[18], [19], [20]]. However, there have been few studies that examine seasonality among Syrian children or refugees [21,22]. In our study, the AOR of consultation for VPD & INF was highest in the winter, whereas the AORs of consultation for NTR, TRA, and NCD & MBD were highest in the summer. Understanding this seasonality can benefit health planners and policymakers in forecasting resource consumption. As some of these illness categories are heterogeneous, a more granular analysis could extend this analysis further.

Strikingly, the effects of forced displacement varied by clinical consultation type. In general, there was a trend towards a “dose-response” effect of displacement, where higher levels of forced displacement correlated with stronger levels of associations (though many confidence intervals did overlap, reducing our ability to distinguish each category fully). The odds of NTR consultation were most notably impacted by displacement, which concurs with existing literature that documents the increased number of children experiencing malnutrition during periods of armed conflict [23]. TRA and NCD & MBD categories also demonstrated increased AORs for clinical consultation during periods of increased forced displacement. At each severity level and time point, the AOR of clinical consultation for TRA was higher than that for NCD & MBD but lower than that for NTR. These trends comport with other research on conflict and health, as conflict is known to cause direct physical injury [4,5]. Conflict also complicates the management of NCDs, many of which require patients to have a steady supply of medications and regular visits with a provider [24].

Unlike NTR, the impact of displacement on AORs for clinical consultation in these categories tended to wane over time (one exception being high levels of displacement for TRA). For NTR however, the effects of displacement did not appreciably decrease. This trend may reflect the magnitude of the malnutrition crisis in NWS [25]. Conversely, there was a dose-response, inverse relationship between VPD & INF consultations and displacement. The AORs modestly trended towards one with increasing time lag, demonstrating that the effects of displacement waned over time (similar to TRA and NCD & MBD). This was surprising, as conflict has been shown to increase the risk of communicable diseases in several other settings [26,27]. While questions around healthcare access may explain the lower AORs of consultation, this is difficult to reconcile with the finding that aside from 2020 (when there were COVID-19-related lockdowns), the number of VPD & INF consultations remained remarkably stable. While there was a slight decrease for months with severe displacement, the monthly number of total consultations was not meaningfully different, further disputing the notion that access to care alone explains these relationships. Further research is needed here to understand this dynamic.

### Limitations

This work has several limitations. SAMS did not collect the consultation data for research purposes, and thus there may have been inaccuracies in data entry or reporting. Some of this potential for error or bias has been reduced by the robust sample size and the subsequent validity checks conducted by the study team. While these data originate from a single NGO, SAMS does carry a sizable footprint in NWS. At the end of 2018, the SAMS facilities included in this study represented over 10% of total health facilities in NWS and approximately 20% of total clinical visits; accordingly, this data reasonably represents the health landscape in NWS. This study only analyses facility-based care, and thus does not adequately capture the full scope of unmet health needs or needs addressed through other means in NWS. While SAMS facilities serve both host and IDP populations, it is reasonable to expect that some communities, especially among IDPs, face significant challenges in accessing health facilities. While this study found that displacement affected clinical utilisation patterns in host and IDP communities, it may underestimate the health needs of the communities that could not access healthcare in conflict settings.

We used OCHA estimates of IDP movements to measure displacement, though they are imperfect. However, the months with the most IDP movements correlated with external estimates of net displacement and to periods with an escalation in armed conflict. In our study, forced displacement may be a reasonable surrogate for conflict intensity, especially as NWS is a densely populated area where increased shelling and armed conflict can be expected to lead to large-scale population movements. Researchers have utilised the magnitude of forced displacement as a proxy for conflict intensity in the Eritrean-Ethiopian conflict and in Colombia [28,29]. However, other researchers have challenged this link, and many additional factors likely impact displacement, such as geography and political or physical restrictions on movement [30].

Lastly, there were fluctuations in the number of reporting facilities throughout our study period. While a total of 51 facilities reported data for this study, our sample began with only 32 facilities, and over the study period, there were 23 facility closures and 20 openings; only 14 sites were operational during the entire 60-month period. Eleven of these 23 closures occurred between May 2019 and March 2020, corresponding with the time of increased regional hostilities. Ma'arrat al-Nu'man district, in the southernmost part of Idlib governorate, showed a 98% decline in overall consultations by December 2019. However, the number of total consultations remained relatively consistent throughout the study period, indicating that these fluctuations likely did not bias our results. Given the proximity of many reporting facilities within each district, it is also reasonable to assume that individuals were able to seek treatment at neighbouring SAMS facilities if a facility was forced to close.

## Conclusion

This large analysis of paediatric health utilisation during Syria's protracted armed conflict adds to the sparse data on this topic. In NWS, forced displacement differentially affects clinical consultations and has lasting impacts on clinical utilisation at least six months after the displacement event. There are implications of this work for aid policy and resource provision. The identified persistent seasonal trends can help health policy planners more effectively anticipate paediatric health needs in NWS, particularly at times of forced displacement. The differences by sex and age can also support more tailored approaches to preventive, promotive, and therapeutic services (e.g., nutritional support for girls, injury prevention programs for boys, and mental health outreach for adolescents). Further research is needed to explore in more depth the barriers and enablers of healthcare access for children and adolescents in NWS, both among IDPs and resident populations. Our research reinforces the need for mental health and psychosocial care, as well as NCD care tailored to paediatric populations. Finally, similar work needs to be undertaken to understand if our findings hold true throughout Syria, regionally, or in broader contexts of conflict.

## Authors' contributions

V Kampalath, MC Tarnas, V Patel, M Hamze, and A Abbara contributed to the conceptualisation, methodology, formal analysis. V Kampalath and V Patel contributed to writing the original draft. R Loutfi, B Tajaldin, A Albik, A Kassas, and A Khashata contributed to the data curation. A Abbara contributed to supervision. All authors contributed to revisions and edits of the manuscript. All authors also had full access to the data in the study and verified the underlying data.

## Funding

None.

## CRediT authorship contribution statement

**Vinay Kampalath:** Writing – review & editing, Writing – original draft, Supervision, Methodology, Investigation, Formal analysis, Data curation, Conceptualization. **Ms Maia C. Tarnas:** Writing – review & editing, Writing – original draft, Visualization, Methodology, Investigation, Formal analysis. **Ms Vaibhavee Patel:** Writing – original draft, Methodology, Data curation, Conceptualization. **Mohamed Hamze:** Writing – review & editing, Writing – original draft, Supervision, Methodology, Investigation, Formal analysis, Conceptualization. **Randa Loutfi:** Writing – review & editing. **Bachir Tajaldin:** Writing – review & editing, Data curation. **Ahmad Albik:** Writing – review & editing, Data curation, Conceptualization. **Ayman Kassas:** Writing – review & editing, Data curation. **Anas Khashata:** Writing – review & editing, Data curation. **Aula Abbara:** Writing – review & editing, Writing – original draft, Supervision, Resources, Project administration, Methodology, Investigation, Formal analysis, Data curation, Conceptualization.

## Declaration of competing interest

All authors declare no competing interests.

## Data Availability

The data used in this study are confidential in nature, and therefore will only be made available upon reasonable request.

## Appendix A Supplementary data

Supplementary material Image 1
