# Supplementary material for: An analysis of paediatric clinical presentations in Northwest Syria and the effect of forced displacement, 2018–2022
Source: Glob Epidemiol. 2024 Jun 4;8:100146. doi: 10.1016/j.gloepi.2024.100146 (PMC11214201; doi:10.1016/j.gloepi.2024.100146)
Supplement: Supplementary file 1 — Supplementary material [file mmc1.pdf]

SUPPLEMENTAL MATERIALS

Model Specification: Random Intercept...…………………………………………….…………1
Unadjusted Odds Ratios………………………..…..……….………………………….…………3
Lagged Model Outputs…..….………………………………………………………………4

Model Specification: Random Intercept

We ran each model with and without a random district-level intercept. For each consultation category at every lag, the models with the random intercept had a lower Akaike Information Criterion (AIC) value. AIC indicates model fit, where a lower number signifies better fit. Tables S1 and S2 show the outputs for each model at no time lag for displacement. We only present these outputs for the sake of brevity, but Table S3 contains a comparison of AIC values for the outputs at each lag tested in this study.

	**VPD & INF**		**NTR**	
	**No random intercept**	**Random intercept**	**No random intercept**	**Random intercept**
	OR (95% CI)	*OR (95% CI)*	*OR (95% CI)*	*OR (95% CI)*
**Displacement**				
Low	Reference			
Medium	0.89 (0.88 - 0.89)	0.83 (0.82 - 0.84)	1.35 (1.28 - 1.41)	1.31 (1.24 - 1.37)
High	0.89 (0.88 - 0.90)	0.85 (0.84 - 0.86)	1.73 (1.64 - 1.83)	1.70 (1.61 - 1.80)
Severe	0.88 (0.87 - 0.88)	0.81 (0.80 - 0.82)	1.52 (1.43 - 1.62)	1.55 (1.46 - 1.66)
**Residency**				
Host	Reference			
Displaced	1.12 (1.11 - 1.12)	1.23 (1.22 - 1.24)	1.10 (1.07 - 1.13)	1.27 (1.23 - 1.31)
**Age (yr.)**				
0 – 1	Reference			
2 – 4	1.28 (1.27 - 1.29)	1.26 (1.26 - 1.27)	0.80 (0.78 - 0.83)	0.84 (0.82 - 0.87)
5 – 11	0.85 (0.84 - 0.85)	0.83 (0.83 - 0.84)	0.36 (0.35 - 0.38)	0.39 (0.37 - 0.40)
12 – 17	0.31 (0.31 - 0.31)	0.31 (0.30 - 0.31)	0.24 (0.22 - 0.25)	0.26 (0.25 - 0.28)
**Sex**				
Male	Reference			
Female	1.01 (1.01 - 1.01)	1.00 (1.00 - 1.01)	1.52 (1.48 - 1.56)	1.52 (1.48 - 1.56)
**Season**				
Winter	Reference			
Spring	0.72 (0.71 - 0.72)	0.73 (0.72 - 0.73)	1.53 (1.46 - 1.59)	1.51 (1.45 - 1.58)
Summer	0.48 (0.48 - 0.49)	0.48 (0.48 - 0.48)	2.02 (1.94 - 2.11)	1.93 (1.85 - 2.01)
Autumn	0.63 (0.63 - 0.64)	0.62 (0.62 - 0.63)	1.60 (1.53 - 1.67)	1.56 (1.50 - 1.63)
**Temporality**	Reference			
Year	0.94 (0.94 - 0.94)	0.92 (0.91 - 0.92)	1.10 (1.08 - 1.11)	1.05 (1.03 - 1.07)
**AIC**	4,127,680	4,073,422	244,611.7	239,467.3

Table S1: Outputs for VPD & INF and NTR consultations.

	TRA		NCD & MBD	
	**No random intercept**	**Random intercept**	**No random intercept**	**Random intercept**
	OR (95% CI)	*OR (95% CI)*	*OR (95% CI)*	*OR (95% CI)*
**Displacement**				
Low	Reference			
Medium	1.10 (1.08 - 1.11)	1.20 (1.18 - 1.21)	1.08 (1.07 - 1.09)	1.10 (1.09 - 1.11)
High	1.07 (1.06 - 1.09)	1.15 (1.13 - 1.17)	1.09 (1.08 - 1.11)	1.11 (1.10 - 1.13)
Severe	1.12 (1.10 - 1.14)	1.21 (1.19 - 1.23)	1.15 (1.14 - 1.17)	1.19 (1.18 - 1.20)
**Residency**				
Host	Reference			
Displaced	0.65 (0.65 - 0.66)	0.65 (0.64 - 0.65)	1.10 (1.10 - 1.11)	1.05 (1.04 - 1.06)
**Age (yr.)**				
0 – 1	Reference			
2 – 4	5.19 (5.09 - 5.29)	5.21 (5.11 - 5.32)	1.35 (1.34 - 1.36)	1.37 (1.35 - 1.38)
5 – 11	8.51 (8.34 - 8.68)	8.47 (8.31 - 8.64)	1.64 (1.63 - 1.65)	1.65 (1.64 - 1.67)
12 – 17	12.72 (12.47 - 12.97)	12.33 (12.09 - 12.57)	1.94 (1.92 - 1.95)	1.94 (1.93 - 1.96)
**Sex**				
Male	Reference			
Female	0.49 (0.49 - 0.50)	0.50 (0.49 - 0.50)	1.14 (1.14 - 1.15)	1.14 (1.14 - 1.15)
**Season**				
Winter	Reference			
Spring	1.28 (1.27 - 1.29)	1.26 (1.24 - 1.27)	1.25 (1.24 - 1.26)	1.25 (1.24 - 1.26)
Summer	1.46 (1.45 - 1.48)	1.47 (1.45 - 1.48)	1.71 (1.69 - 1.72)	1.70 (1.69 - 1.72)
Autumn	1.39 (1.38 - 1.41)	1.41 (1.40 - 1.43)	1.37 (1.36 - 1.38)	1.37 (1.36 - 1.38)
**Temporality**	Reference			
Year	1.08 (1.07 - 1.08)	1.14 (1.14 - 1.15)	1.03 (1.03 - 1.03)	1.03 (1.03 - 1.03)
**AIC**	2,054,419	2,007,213	3,484,271	3,471,894

Table S2: Outputs for TRA and NCD & MBD consultations.

Akaike Information Criterion (AIC)					
	**No random intercept**	**Random intercept**		**No random intercept**	**Random intercept**
**VPD & INF**			**TRA**		
1 month	4,070,654	4,016,766	1 month	2,028,477	1,981,724
6 months	3,759,327	3,706,948	6 months	1,879,669	1,833,554
**NTR**			**NCD & MBD**		
1 month	242,434.7	237,302	1 month	3,439,967	3,427,557
6 months	228,451.4	223,048.4	6 months	3,179,489	3,167,085

Table S3: AIC values for the remaining models (lagged at one and six months)

Unadjusted Odds Ratios

Table S4 contains the unadjusted odds ratios for consultations in each category. Each model contained a random district-level intercept.

	VPD & INF	NTR	TRA	NCD & MBD
	OR (95% CI)	*OR (95% CI)*	*OR (95% CI)*	*OR (95% CI)*
**Displacement**				
Low	Reference			
Medium	0.86 (0.85 - 0.87)	1.29 (1.24 - 1.35)	1.02 (1.01 - 1.03)	1.13 (1.12 - 1.14)
High	0.99 (0.99 - 1.00)	1.55 (1.49 - 1.61)	0.86 (0.85 - 0.87)	1.06 (1.05 - 1.07)
Severe	1.03 (1.03 - 1.04)	1.35 (1.29 - 1.41)	0.85 (0.84 - 0.86)	1.08 (1.08 - 1.09)
**Residency**				
Host	Reference			
Displaced	1.29 (1.29 - 1.30)	1.36 (1.32 - 1.41)	0.62 (0.61 - 0.62)	1.00 (1.00 - 1.01)
**Age (yr.)**				
0 – 1	Reference			
2 – 4	1.24 (1.23 - 1.25)	0.84 (0.81 - 0.87)	5.29 (5.19 - 5.39)	1.37 (1.36 - 1.38)
5 – 11	0.81 (0.81 - 0.82)	0.38 (0.37 - 0.40)	8.73 (8.56 - 8.90)	1.66 (1.65 - 1.67)
12 – 17	0.30 (0.29 - 0.30)	0.26 (0.25 - 0.28)	12.46 (12.22 - 12.71)	1.98 (1.96 - 1.99)
**Sex**				
Male	Reference			
Female	0.97 (0.96 - 0.97)	1.46 (1.42 - 1.50)	0.53 (0.53 - 0.53)	1.15 (1.15 - 1.16)
**Season**				
Winter	Reference			
Spring	0.72 (0.71 - 0.72)	1.53 (1.46 - 1.59)	1.29 (1.28 - 1.30)	1.27 (1.26 - 1.28)
Summer	0.47 (0.47 - 0.48)	1.88 (1.81 - 1.96)	1.56 (1.54 - 1.57)	1.73 (1.72 - 1.74)
Autumn	0.64 (0.63 - 0.64)	1.52 (1.46 - 1.59)	1.41 (1.40 - 1.43)	1.36 (1.35 - 1.37)
**Temporality**	Reference			
Year	0.98 (0.98 - 0.98)	0.95 (0.94 - 0.96)	1.07 (1.07 - 1.08)	0.99 (0.99 - 1.00)

Table S4: Unadjusted odds ratios with 95% confidence intervals.

Lagged Model Outputs
To assess the associations between displacement and consultations over time, we lagged the displacement variable one and six months. Table 3 in the main text reports the AORs for the displacement categories across each lag. Tables S5 and S6 report the full outputs of each model.

One Month Lag				
	**VPD & INF**	**NTR**	**TRA**	**NCD & MBD**
	OR (95% CI)	*OR (95% CI)*	*OR (95% CI)*	*OR (95% CI)*
**Displacement**				
Low	Reference			
Medium	0.81 (0.80 - 0.81)	1.43 (1.37 - 1.50)	1.18 (1.17 - 1.20)	1.14 (1.13 - 1.15)
High	0.85 (0.84 - 0.86)	1.56 (1.47 - 1.65)	1.23 (1.21 - 1.25)	1.08 (1.06 - 1.09)
Severe	0.85 (0.84 - 0.85)	1.51 (1.42 - 1.61)	1.19 (1.17 - 1.21)	1.15 (1.14 - 1.16)
**Residency**				
Host	Reference			
Displaced	1.23 (1.23 - 1.24)	1.28 (1.24 - 1.32)	0.64 (0.64 - 0.65)	1.05 (1.04 - 1.05)
**Age (yr.)**				
0 – 1	Reference			
2 – 4	1.27 (1.26 - 1.27)	0.85 (0.82 - 0.88)	5.23 (5.13 - 5.34)	1.37 (1.36 - 1.38)
5 – 11	0.84 (0.83 - 0.84)	0.39 (0.37 - 0.40)	8.52 (8.35 - 8.69)	1.65 (1.63 - 1.66)
12 – 17	0.31 (0.30 - 0.31)	0.26 (0.24 - 0.27)	12.35 (12.11 - 12.60)	1.93 (1.92 - 1.95)
**Sex**				
Male	Reference			
Female	1.00 (1.00 - 1.01)	1.52 (1.48 - 1.56)	0.50 (0.49 - 0.50)	1.14 (1.14 - 1.15)
**Season**				
Winter	Reference			
Spring	0.71 (0.70 - 0.71)	1.54 (1.48 - 1.61)	1.29 (1.28 - 1.31)	1.26 (1.25 - 1.27)
Summer	0.48 (0.48 - 0.48)	1.88 (1.81 - 1.96)	1.48 (1.47 - 1.50)	1.69 (1.68 - 1.70)
Autumn	0.64 (0.63 - 0.64)	1.56 (1.49 - 1.62)	1.39 (1.38 - 1.41)	1.35 (1.34 - 1.36)
**Temporality**	Reference			
Year	0.92 (0.92 - 0.92)	1.03 (1.02 - 1.05)	1.15 (1.14 - 1.16)	1.02 (1.02 - 1.03)

Table S5: Adjusted odds ratios with 95% confidence intervals for a one-month displacement lag.

Six Month Lag				
	**VPD & INF**	**NTR**	**TRA**	**NCD & MBD**
	OR (95% CI)	*OR (95% CI)*	*OR (95% CI)*	*OR (95% CI)*
**Displacement**				
Low	Reference			
Medium	0.87 (0.87 - 0.88)	1.36 (1.30 - 1.43)	1.11 (1.09 - 1.12)	1.08 (1.07 - 1.09)
High	0.88 (0.87 - 0.89)	1.60 (1.50 - 1.70)	1.23 (1.21 - 1.25)	1.04 (1.03 - 1.05)
Severe	0.86 (0.86 - 0.87)	1.57 (1.48 - 1.66)	1.15 (1.13 – 1.17)	1.08 (1.07 - 1.10)
**Residency**				
Host	Reference			
Displaced	1.27 (1.27 - 1.28)	1.25 (1.21 - 1.29)	0.63 (0.62 - 0.63)	1.05 (1.04 - 1.05)
**Age (yr.)**				
0 – 1	Reference			
2 – 4	1.26 (1.25 - 1.27)	0.84 (0.81 - 0.87)	5.33 (5.22 - 5.44)	1.37 (1.36 - 1.39)
5 – 11	0.84 (0.83 - 0.84)	0.38 (0.36 - 0.40)	8.67 (8.49 - 8.85)	1.65 (1.63 - 1.66)
12 – 17	0.31 (0.30 - 0.31)	0.25 (0.24 - 0.27)	12.54 (12.28 - 12.80)	1.95 (1.93 - 1.97)
**Sex**				
Male	Reference			
Female	1.01 (1.00 - 1.01)	1.53 (1.49 - 1.58)	0.50 (0.49 - 0.50)	1.14 (1.14 - 1.15)
**Season**				
Winter	Reference			
Spring	0.71 (0.71 - 0.72)	1.58 (1.51 - 1.65)	1.29 (1.27 - 1.30)	1.26 (1.25 - 1.27)
Summer	0.45 (0.45 - 0.46)	2.04 (1.96 - 2.14)	1.55 (1.53 - 1.57)	1.76 (1.74 - 1.77)
Autumn	0.61 (0.61 - 0.62)	1.57 (1.50 - 1.64)	1.40 (1.38 - 1.42)	1.39 (1.37 - 1.40)
**Temporality**	Reference			
Year	0.92 (0.92 - 0.93)	1.01 (0.99 - 1.03)	1.15 (1.14 - 1.15)	1.01 (1.01 - 1.02)

Table S6: Adjusted odds ratios with 95% confidence intervals for a six-month displacement lag.
